# Maximizing biomedical research impacts through bioethical considerations

**DOI:** 10.1242/dmm.050046

**Published:** 2023-04-24

**Authors:** Valerie A. Tornini, Santiago Peregalli Politi, Lori Bruce, Stephen R. Latham

**Affiliations:** ^1^ Department of Genetics, Yale School of Medicine, New Haven, CT 06511, USA; ^2^ Child and Adolescent Psychiatry Clinical Services, Geneva University Hospital, Geneva 1205, Switzerland; ^3^ Interdisciplinary Center for Bioethics, Yale University, New Haven, CT 06511, USA

## Abstract

Bioethics is the formal study of ethical judgments concerning the advances and applications of biology, medicine and related technologies. In a time of unprecedented biomedical advances, it is critical to integrate bioethical frameworks more fully into biomedical research to align these scientific advances with their intended societal needs. In this Perspective, we describe some motivations and frameworks for cross-disciplinary bioethical training for biomedical researchers, and discuss how actively considering bioethics in research and study design could maximize biomedical researchers' intended impacts in society.

## Teaser

**Summary:** Integrating bioethical frameworks and methodologies with biomedical research can have a key positive influence on the research's societal outcomes.

## Introduction

The pursuit of scientific discovery at the leading edge carries with it inescapable and ever-evolving bioethical challenges. Broadly, bioethics is the study of the ethical issues – the discernment and application of the concepts of right and wrong, value and disvalue – that arise from advances in and applications of biology, medicine and related technologies. The act of doing scientific research both raises and informs ethical issues.

As we continue to push boundaries with questions related to the human experience, scientific breakthroughs carry with them some of the most pressing bioethical considerations of our time. It is becoming more difficult to consider the vast impacts that biological discoveries have on a simultaneously hyperspecialized, yet interdisciplinary world. How can biomedical researchers maximize good (beneficence), while doing the least harm (non-maleficence)? How can researchers and physicians frame and understand ‘good’ and ‘harm’, who do those definitions center, who should they center, and who should be at the forefront of asking and confronting these bioethical issues? Furthermore, how can researchers overcome barriers – many of them related to finite resources – to embedding bioethical considerations within their education and research? Addressing these kinds of questions requires researchers to develop and understand their own ethical intentions that comprise the values and reasonings behind actions, as well as ethical theories or frameworks that can guide them in maximizing the impact of their research (Box 1).

**Box 1. Bioethical approaches and branches**

**Normative ethics:** a branch of ethics concerned with criteria of what is morally right and wrong.

**Metaethics:** a branch of ethics concerned with understanding the assumptions underlying theories of normative ethics.

**Applied ethics:** a branch of ethics concerned with the application of ethics to real-world problems.

**Principlism:** an ethical approach commonly used in healthcare and biomedical research, which emphasizes four key ethical principles: respect for autonomy, beneficence, non-maleficence and justice (Beauchamp and Childress, 2013). Other variations of principlism exist.

**Casuistry:** a practical, case-based process of reasoning that seeks to resolve ethical questions through applying or extending principles and values developed through interpretation of settled cases to novel cases.

**Community ethics:** a means of amplifying diverse voices and values of community members within institutional and public policy related to biomedical research and clinical care (Bruce et al., 2022).

**Feminist/care ethics:** a normative ethical theory that centers interpersonal relationships and care or benevolence on moral actions. This theory places importance on the response to an individual. It also challenges some patriarchal systems that valorize caring as a feminine quality and place undue burdens typically on women, instead of placing the importance of caring across individuals regardless of gender.

**One Health:** a multisectoral, collaborative, transdisciplinary approach, working across scales, that works to attain optimal health for people, animals and the environment. Key elements of this approach include geographical, ecological, human activities and food–agricultural components.

**Decolonial and Indigenous bioethics:** theories and approaches that challenge the assumptions and consequences of coloniality, and generally favor intercultural and pluriversal frameworks.

**Sustainable development ethics:** ethical approaches to how human should live in relation to other persons and communities, to the natural world, and to current and future generations.

**Trauma-informed care:** an evidence-based approach that asks what's ‘going on’ for someone instead of what's ‘wrong’ with them; the approach centers the physical and emotional safety of all involved individuals (e.g. scientists, legislators, impacted populations, patients and clinicians) and acknowledges the impacts of trauma on all aspects of research, law, health and healthcare. This approach systematically considers culture, history, power dynamics and marginalization, among other factors, that contribute to psychosocial effects of trauma on an individual (Substance Abuse and Mental Health Services Administration, 2014) and has been applied to clinical ethics consultations (Lanphier and Anani, 2022).

**Trauma-informed policymaking**: an evidence-based approach that applies trauma-informed principles to institutional policymaking, which can be applied to research institutions, hospitals, and other private and public organizations (Bruce and Herbst, 2022).

**Dialectics:** the process or discourse of two or more different or contradictory views about a subject, but wishing to establish a truth through reasoned and logical argumentation (Linehan, 1993).

**Good Participatory Practice (GPP) guidelines:** guidelines for all interested parties or partners, including those affecting research conduct, funding and sponsorship, as well as those who may be affected by it, to contribute to accomplishing a project's or trial's aims. These guidelines were originally established by the Joint United Nations Programme on HIV/AIDS (UNAIDS) and the AIDS Vaccine Advocacy Coalition (AVAC), and have been further adapted to other contexts (AVAC and UNAIDS, 2011; Wilson et al., 2021).

**Patient and Public Involvement (PPI) guidelines:** guidelines for researchers and investigators to involve the public, patients and other service users in their research, to ensure that the needs and expectations of those most affected are met to the greatest extent possible (Pollard et al., 2015; PPI Resources for Applicants to NIHR Research Programmes).

In this Perspective, we briefly discuss some motivations and frameworks for implementing cross-disciplinary bioethical training into biomedical research, and an outlook on how such implementation could maximize biomedical researchers' intended societal impacts (Chambers, 2016).

“It is becoming more difficult to consider the vast impacts that biological discoveries have on a simultaneously hyperspecialized, yet interdisciplinary world.”

## Barriers to bioethics integration with biomedical research

Just like biomedical research capacities across different countries, bioethical training, research ethics reviews and research ethics committees also vary widely across different countries, with challenges associated with research ethics, and training in low- and middle-income countries being even more complex (Hyder et al., 2009; Pratt and Hyder, 2015; Gopichandran, 2017). Generally, in biomedical research, there is minimal coursework or training related to the formal study of bioethics in biomedical research curricula beyond a focus on responsible research integrity and the protection of human and animal subjects (Box 2), as founded in the Declaration of Helsinki and the Belmont Report. Such coursework tends to focus less on intriguing ethical dilemmas and robust ethical frameworks, opting instead to fulfill regulatory requirements. These prescriptive guidelines and regulations sometimes fail to fully engage biomedical researchers, and often feedback on, or understanding of, these guidelines from researchers is not evaluated.

**Box 2. International Research Integrity guidelines**

There are various bioethical guidelines across countries that aim to help biomedical researchers align their research with bioethical regulation. In the United States, the ‘Responsible Conduct of Research requirements’ (RCR) encompass most of the required training for federally funded research (NIH, 2022), some of which is required to be completed before researchers can perform their work. Similarly, researchers in the United Kingdom follow the Concordat to Support Research Integrity and the Singapore Statement on Research Integrity, as well as strict ethical animal use guidelines. Furthermore, the Indian Council of Medical Research provides national ethical guidelines for researchers, and partnerships through the United Nations Educational, Scientific and Cultural Organization (UNESCO) are potentiating intranational and international bioethics guidance globally, including in Latin America and the Caribbean and in Africa (Redbioética/UNESCO, 2019; UNESCO Intergovernmental Bioethics Committee; Evaluation of UNESCO's Bioethics and Ethics of Science and Technology Programme). Moreover, the European Network for Research Ethics and Integrity (ENERI) Project has the important role of gathering global research ethics codes and guidelines. It is worth noting that different subfields may have more specific guidelines. For example, bioethical guidelines can be tailored for research that involves vertebrate or invertebrate animals, human samples, or patients and/or healthy participants, particularly those from minority populations, such as Indigenous people or migrants (Anekwe, 2015; Claw et al., 2018; Deps et al., 2022).

This leads to numerous challenges to fully integrating bioethical training into biomedical research training. Already compacted curricula in biomedical and medicine programs allow little time and flexibility for pedagogical integration of bioethics. There is also a nuanced balance of depth versus breadth of bioethics training, which can often lead to the perception that specific areas of training are being neglected, impacting scientific or medical rigor. Further, there are often structural barriers between existing bioethics programs and biomedical research programs, even within single institutes (Wilson et al., 2021; AVAC and UNAIDS, 2011). More broadly, differing viewpoints on prioritization of bioethics and their subfields, including inherently different cultural perspectives across different countries or backgrounds, challenge the notion of a universal bioethics curriculum. Lastly, there is a perception that biomedical training and research should focus on the scientific ‘is’ and ‘can’, rather than the more bioethical ‘should’ and ‘ought’ (Mathews et al., 2016). Within the societal systems that many scientific enterprises operate, the forces of capitalism and competition – especially those driven by economic and political forces for private profit – exist in tension with the values that bioethics strives to address in biomedical research, such as respect for autonomy, beneficence and privacy. These tensions, along with those that exist between time, resources, scientific output and bioethical considerations, must be reconciled in ways that enhance the societal benefit of biomedical research.

Despite these challenges, we posit that a holistic integration of such training could lead to short- and long-term societal benefits. Researchers engaged in ‘Responsible Conduct of Research requirements’ (RCR) training, or international equivalents, could benefit by shifting away from perceiving training as a regulation compliance, and instead develop an understanding and appreciation of these regulations' underlying ethical ideals that will help them develop their own praxis to integrate their work with ethical decision making (Anderson, 2016).

“Within the societal systems that many scientific enterprises operate, the forces of capitalism and competition – especially those driven by economic and political forces for private profit – exist in tension with the values that bioethics strives to address in biomedical research, such as respect for autonomy, beneficence and privacy.”

## Interdisciplinary bioethics training can enhance study design

Despite our different career stages and professions (Box 3), we met through a mutual desire to understand and shape the broader impacts of our work. A deep foray into bioethics exposed us to frameworks to help shape our research and medical pursuits in line with their intended societal impacts. We explored bioethics beyond our specialized contexts through an interdisciplinary bioethics summer institute (Sherwin B. Nuland Summer Institute in Bioethics). This immersion allowed us to glimpse a truly interdisciplinary world and explore how bioethical frameworks could be used to synergize professional pursuits to maximize beneficence.

**Box 3. Cross-disciplinary approaches to bioethics – authors' expertise**

**Valerie Tornini, PhD** is a postdoctoral researcher at Yale School of Medicine, studying the genetic basis of cell specification and their implications in developmental disorders.

**Santiago Peregalli Politi, MD, MBE** is a child psychiatrist and psychotherapist at Geneva University Hospitals, and a faculty member of the Summer Institute in Bioethics at Yale's Interdisciplinary Center for Bioethics and of the Observatory on Bioethics and Law at Universitat de Barcelona.

**Lori Bruce, MA, MBE, HEC-C** is a bioethicist, the Associate Director of Yale's Interdisciplinary Center for Bioethics, Founder and Chair of the Community Bioethics Forum at Yale University, Co-Chair of the Adult Ethics Committee at Yale-New Haven Hospital, and Director of the Summer Institute in Bioethics at Yale's Interdisciplinary Center for Bioethics. Her work focuses on ethical policymaking, the ethics of psychedelics, the amplification of community voices and values within policymaking, and consent for intimate (i.e. pelvic and prostate) examinations.

**Stephen R. Latham, JD, PhD** is the Director of Yale's Interdisciplinary Center for Bioethics. He teaches and writes about bioethics, health law and environmental ethics.

To understand how researchers can incorporate bioethics into their work, we draw a parallel between bioethics and dialectical methods. With a goal of establishing a truth through reasoned arguments, dialectics is the idea that opposite emotions or thoughts can co-exist. Both could be true, even if, from a logical perspective, we would have difficulties understanding that opposite emotions or thoughts are both partially true. Dialectics are used in different areas in order to add flexibility to our understanding and thinking, as in pedagogy (Shea, 2020) or psychiatry and psychotherapy.

In dialectical behavior therapy (Linehan, 1993), a prominent example is used, in which a person is walking along a river, and a passerby pushes them into the river. This person did not do anything to find themself in the river, yet it is their responsibility to get out of the river in order to not drown (Miller et al., 2006). If ethical dilemmas are that river in which researchers find themselves, it is also their duty to responsibly grapple with those problems and solutions, taking into account diverse and sometimes dialectically opposed viewpoints. As one practical example, recent advances in genetically modified mouse embryoids have produced models that do not fully form a brain when development is stopped at an early timepoint (Amadei et al., 2022; Tarazi et al., 2022). This, in itself, is a creative scientific solution to help avoid ethical issues of generating sentient and thinking embryoids, but, opposingly, it is also true that it creates research, disease modeling and therapeutic constraints (Greely, 2021; MIT Technology Review, 2022). This clearly poses a dilemma for researchers who are concerned with ethical issues regarding animal rights but who are also keen to pursue scientific progress. Should they use a model system with these scientific limitations? To what extent are these embryoids simultaneously animals and not animals? Are there complementary model systems that could be used to overcome these limitations, which together can build stronger evidence for a biological phenomenon? Could existing frameworks or bioethical methodologies be adapted for mouse embryoid research (for example, Park et al., 2017)? And, what are the responsibilities, not only of the researchers but of other colleagues and the public, in ensuring ethical conduct and thoughtfulness? A deeper understanding of bioethics, or in this scenario, specifically dialectics, could help researchers accept and interpret these opposing, and sometimes difficult, truths and ultimately make informed decisions on study design.

Thus, bioethics, as a multidisciplinary field that considers ethical implications along a vast spectrum, gives us a great opportunity to inject flexibility into our thinking, and it helps give us an understanding of paradoxical situations in which two truths could appear to be irreconcilable. Bioethics, more generally, gives us tools to identify, examine and resolve ethical tensions within biomedical research as well as within medicine, law, public health, policymaking, and so forth. Scientists armed with dialectical and bioethical tools would be able to fully ensure that their work is informed by ethical guidelines and is aligned with societal values.

## How to integrate bioethics with biomedical research

**Fig. 1.**
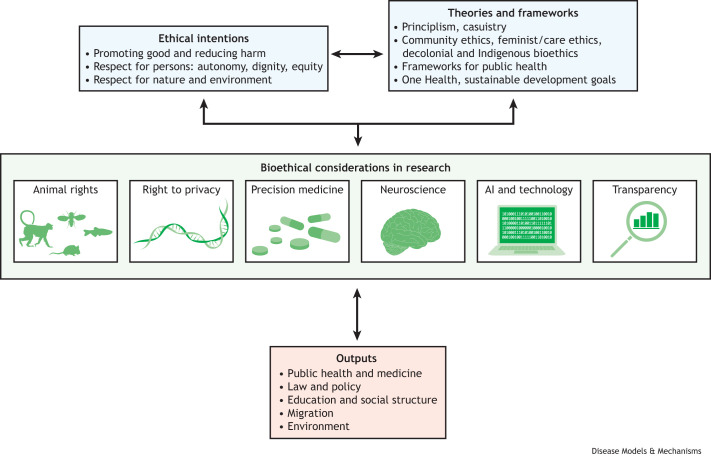
**Bioethical considerations for biomedical researchers.** Various ethical intentions (top left) and bioethics theories and related frameworks (top right) can influence bioethical considerations in biomedical research (middle), ultimately affecting their systemic (or societal) outputs (bottom). The ethical intentions that relate to respect for persons include racial equity, Indigenous and migrant rights, gender diversity, cultural and religious diversity, and representation for people with disabilities or rare diseases, and patients with a range of ages, among many others. We invite the reader to consider how any one of the fields shown in the figure, and others not included, may be linked with their research work, and how different bioethical considerations can shape and reshape their intended impacts. AI, artificial intelligence.

Moving forward, there are many approaches to address the challenges in embedding bioethics within biomedical research. These approaches can lead to major impacts on societal outputs such as public health, law and policy, education, migration, social structures or systems, medicine and medical care, and the environment (Fig. 1). Scientists and clinicians can, of course, study bioethics, but they can also collaborate and/or partner with bioethicists when designing their experiments and reviewing their findings. For example, bioethicists were incorporated into study design and analysis of a recent study on restoring circulation and cellular functions in pig brains after death (Vrselja et al., 2019; The New York Times, 2022). More broadly, they can partner with bioethicists, such as those in underutilized ethics initiatives, to develop ethical guidelines for their subfield that could even be sponsored by their medical or scientific associations, like the World Health Organization guidelines that outline ethical boundaries and governance mechanisms for human genome editing, among other examples (European Commission Ethics Guidelines for Trustworthy Artificial Intelligence). Embracing collaboration can also break down structural barriers between departments and institutes. Researchers can also partner with community ethics committees (CECs), which are advisory groups consisting of diverse community members with basic bioethics education who learn from and engage with speakers (such as experts in the field, advocates or affected persons), reflect on ethical challenges, and then share their perspectives on health and scientific dilemmas and advances. Members are diverse in age, cultural affiliation, religion, occupation and political leaning. They are also often impacted by biomedical research and health policies and, as such, can speak to the complexities of how the research community can rebuild public trust in science, and how we can foster increased transparency and empathy within research and medicine. These CECs can incorporate varying perspectives that consider different cultures when tackling bioethical issues. The work of CECs has informed scores of policies over the past decade that are directly informed by biomedical research, such as policies governing guidance on physician-assisted suicide (Powers and McLean, 2011), brain death (Neurocritical Care Society), and coronavirus disease 2019 (COVID-19) research and allocation protocols (Tolchin et al., 2020). Lastly, successful interdisciplinary partnerships or collaborations should consider methodologies and fields outside their own as equally rigorous and legitimate, and should align research questions and approaches with other disciplines' suggestions or contributions. Table 1 presents some bioethics organizations through which scientists can find more resources.

**Table 1.**
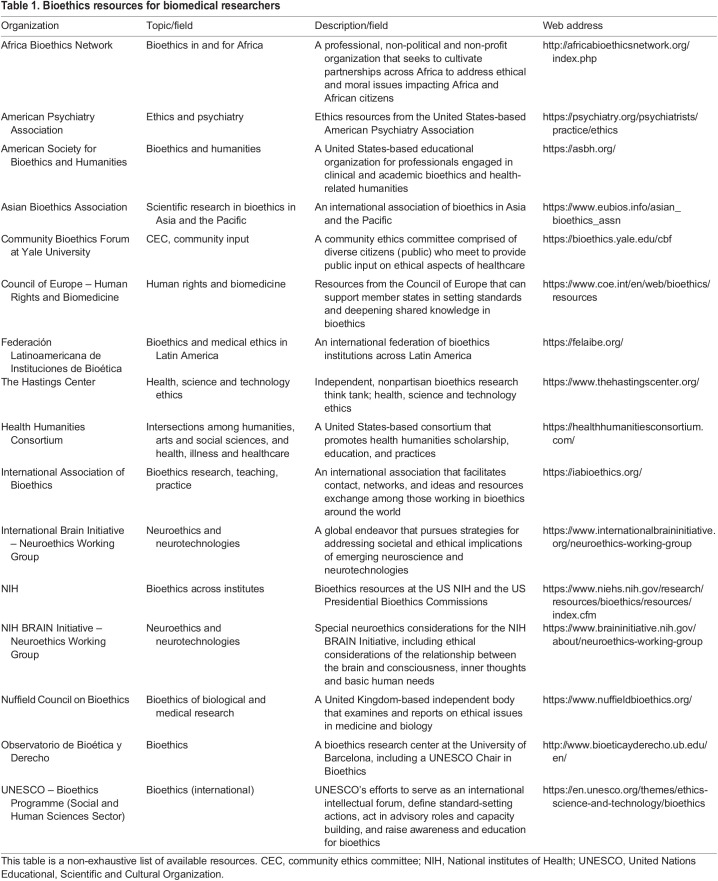
Bioethics resources for biomedical researchers

Funding opportunities are helping support efforts to prioritize bioethics in biomedical research (Sharp et al., 2008). For example, fully funded postbaccalaureate and postdoctoral fellowships and funding awards in bioethics are available through the US National Institutes of Health (NIH) Department of Bioethics, through the UK Institute of Medical Ethics, or through organization- or university-sponsored centers and institutes (The Hastings Center; European Association of Centres of Medical Ethics). The NIH recently presented a symposium on how these efforts have successfully integrated bioethics into biomedical research projects. The Dana Foundation, a private United States-based philanthropic organization, funds grants that support cross-disciplinary projects at the intersection of neuroscience and ethics, policy, law, arts and humanities. Finally, including bioethicists on research institutes' board of directors or advisory teams, as done in the Allen Institute, can further support prioritizing bioethics in research endeavors.

There are many strategies that can be harnessed to holistically integrate bioethics in biomedical research, which has the potential to benefit society, as a whole. However, implementation of this is futile if bioethics does not carefully consider the impact on diverse communities.

“Yet, [bioethical approaches] also invite – and necessitate – researchers to more critically assess how their science is done, to scrutinize how bioethics has been used to mask or enable practices that harm marginalized persons or communities, and to implement strategies across disciplines and communities to use bioethics as a critical tool for positive social change.”

## Embracing diverse communities to shape bioethics

Bioethical frameworks that prioritize inclusion and amplify community values can help reduce the risk of pathologizing inherent parts of a biologically diverse society, for example, creating health disparities between people of different races, genders, sexual orientations, mental or physical abilities, and/or their intersections. Researchers working together with communities who are neurodiverse, gender diverse or racially diverse, and with members who have disabilities, for example, can reshape biological education and research that encompasses natural human diversity, and can directly impact social outputs that center the people in those communities (Danis et al., 2016; Global Neuroethics Summit Delegates et al., 2018; Miyagi et al., 2021; Reynolds and Wieseler, 2022).

Despite societal differences in backgrounds, cultures, expertise and perspectives, bioethical considerations in research can help build a better world by taking into account the spectrum of diverse human experiences. We view it as an ethical responsibility for researchers to seek out these intersecting bioethical areas of basic and biomedical research, and to work with different professional and social communities to synergize research impacts and capacity building, for example, through reference to Patient and Public Involvement (PPI) and Good Participatory Practice (GPP) guidelines (Box 1).

An example of one of the most urgent bioethical imperatives that affects everyone, and that disproportionately affects marginalized populations, is climate change and environmental emergencies. There is an acknowledgement of the extraordinarily high carbon footprint and waste produced by research laboratories, which some individual laboratories are attempting to address (Monaghan et al., 2023). Beyond this laboratory-produced carbon footprint, climate change directly impacts health security. It affects disease susceptibility, onset, progression and access to treatment across the world; uproots communities through forced migration; and exacerbates poverty, food insecurity and political instability – all while disproportionately affecting the most vulnerable and systemically excluded or marginalized. Ongoing efforts, like those in environmental engineering that center research for and with historically marginalized communities, could be adapted to biomedical research contexts (Masten et al., 2021). By more fully integrating ethical values and frameworks into their research plans (as in Fig. 1), researchers and clinicians can better understand, adapt to and develop strategies for addressing emerging biological and health problems that arise from the climate crisis. They can focus capacity building and research, preventative measures and treatments more closely related to these communities' needs, and they can better work together with the communities they aim to serve (Johnson and Wahlert, 2019).

“[…] we view bioethics not as a framework to police or constrain science, but instead as a means of creatively considering dilemmas, welcoming differences and understanding social evolution through a multidisciplinary prism.”

## Outlook

Global societies often hyperfocus on extreme and absolute truths (Wilson et al., 2020; Uba and Bosi, 2022). Therefore, we have discussed how dialectics and other bioethical approaches can be useful to increase mental flexibility by considering paradoxical thoughts relevant to biomedical research and connecting diverse approaches. Yet, they also invite – and necessitate – researchers to more critically assess how their science is done, to scrutinize how bioethics has been used to mask or enable practices that harm marginalized persons or communities, and to implement strategies across disciplines and communities to use bioethics as a critical tool for positive social change.

This Perspective provides a few non-exhaustive snapshots of bioethics in biomedical research contexts. In Fig. 1, we present a visualization for contextualizing some broad bioethical considerations. What other connections could the reader relate to their own work? Integrated into biomedical research, we view bioethics not as a framework to police or constrain science, but instead as a means of creatively considering dilemmas, welcoming differences and understanding social evolution through a multidisciplinary prism. In this way, bioethics can be a key influence on the societal outcomes of biomedical research.
